# Sexual and mental health of Singaporean gay, bisexual and other men who have sex with men in times of COVID-19: a qualitative study

**DOI:** 10.1080/17482631.2024.2408816

**Published:** 2024-10-10

**Authors:** Xin Yi Seah, Rayner Kay Jin Tan, Xu Ming Yong, Miho Asano

**Affiliations:** a Saw Swee Hock School of Public Health, National University of Singapore and National University Health System, Singapore, Singapore; b University of North Carolina Project-China, Guangzhou, China; c Faculty of Health and Social Development, University of British Columbia

**Keywords:** GBMSM, COVID-19, mental, sexual, well-being, access, health services

## Abstract

**Objectives:**

COVID-19 had significant influences on everyone’s lives. This study aimed to explore impacts of COVID-19 on mental and sexual health and access to health services among gay, bisexual and other men who have sex with men (GBMSM) in Singapore.

**Methods:**

This qualitative study recruited 16 self-identified GBMSM via purposive sampling and semi-structured individual interviews were conducted. Three themes and seven sub-themes were derived from analysis done using the framework method.

**Results:**

Participants shared how COVID-19 led to negative emotions and experiences at an intrapersonal level and interpersonal level (with families or partners), which were also worsened by prevailing stigma that GBMSM already face in Singapore and within their social networks. Sexual behaviours associated with HIV and other sexually transmitted infections risk and substance use were seen to be maladaptive coping methods of social isolation due to COVID-19. These dynamics were all exacerbated by the closure of “non-essential” services, which included many important services for mental and sexual health that were relevant to the GBMSM community.

**Conclusions:**

Changes in policies and community efforts should be explored to improve these areas, enhancing the psychosocial and sexual well-being of GBMSM.

## Introduction

As part of responses to stem the spread of COVID-19 when the pandemic first started in 2020, countries including Singapore rolled out several measures in the hope of mitigating the pandemic. Restrictive measures such as minimal interaction with people beyond households or temporary closure of health or social services deemed non-essential were implemented. In April 2020, a lockdown termed circuit-breaker (CB) was announced by the Singapore government (Gov.sg, 2020). The CB was the first and the most stringent part of Singapore’s lockdown measures, aimed to arrest ascending cases of COVID-19 by reducing social interaction. Work duties were also shifted home, if possible, to minimize social interactions beyond homes and only services defined as essential were allowed operations. Three different phases with different social distancing measures were implemented post-circuit breaker, starting on 2 June 2020. This phase approach enabled Singapore to resume services and activities in a safe and controlled manner (Gov.sg, 2020). However, while tackling COVID-19, authorities could have resulted in broadening inequalities in minority groups, who are initially already facing disparities in health services.

The topic of the vulnerabilities of sexual minorities has been discussed in various aspects such as health, religion, and societal policies. GBMSM represent a pivotal demographic, markedly impacted by increased vulnerabilities in sexual and mental health. Research consistently reveals that these men experience more significant health-related vulnerabilities and detrimental health outcomes compared to heterosexual men. This disparity is evident in the increased prevalence of mental health concerns among GBMSM encompassing a higher incidence of suicidal ideation and behaviours, as well as instances of non-suicidal self-harm (McGarty et al., 2021; Tan et al., 2021a).

Globally, several countries had already legalized same-sex marriages and showed acceptance to the gay, bisexual and queer men who have sex with men (GBMSM) community, but not in Asia (Hsu & Yen, 2017). Although campaigns and organizations have been spreading awareness about GBMSM, the majority is still not accepting towards this community. GBMSM report unfair treatment in different aspects of their lives (e.g., work, family, policies). Not only is such discrimination detrimental to their physical and mental health but will also negatively affect the society (Centers for Disease Control and Prevention CDC, 2016).

In the Singapore context, penal code section 377A, which criminalized sex between consenting adult men, was only recently repealed in December 2022 (BBC News, 2022). Section 377A had been in Penal Code since 1938 and, for several decades, numerous advocates had tried to appeal for it to be repealed but were unsuccessful till recent years. This reflected the conservative mindset of the country towards GBMSM and how a family unit should be represented by a male, female and children. Even though the discriminating section 377A was repealed, the government paid attention to highlight that policies will still protect heterosexual couple families, thus showing that the country is still not accepting towards GBMSM (Today Online, 2022). Legislation regarding criminal acts directed at sexual minorities has conceivably played a part in fostering these adverse attitudes in society.

COVID-19 affected many lives in various aspects, while prolonged restrictive measures added to the existing mental burden people were already facing. Based on the Minority Stress Theory, people from minority groups experience different forms of discriminatory events regularly, which can lead to negative mental health outcomes (Meyer, 2013). In line with the minority stress theory, it was reported within LGBTQ+ populations that elevated rates of depression, anxiety and self-harming behaviours were observed, as compared to their heterosexual or cisgender counterparts (Perez-Brumer et al., 2017). Stress experienced may lead to risk-taking behaviours like substance abuse or engaging in unprotected casual sex (Amaro et al., 2021).

Circumstances of quarantine have been linked to heightened occurrences of mental health issues, exacerbated by factors such as the length of quarantine, apprehension of contracting the disease, feelings of annoyance, lack of sufficient information, financial setbacks and societal stigma (Brooks et al., 2020). These strains, compounded by additional anxieties and fears associated with the pandemic, are expected to escalate levels of anxiety (Rubin & Wessely, 2020) and depression (Brooks et al., 2020; Holmes et al., 2020) among the population.

GBMSM tend to depend on their own community for support, due to the discrimination they face. During the CB period in Singapore, bars and clubs were temporarily closed to minimize social interactions. Bars and clubs that catered specifically to GBMSM usually provide a safe environment for GBMSM, as they were allowed to express themselves freely within these spaces and were able to connect with the other GBMSM without being discriminated against (Croff et al., 2017). In the Singapore context, as the country is not readily accepting towards GBMSM, there are few known support groups that are openly publicized to GBMSM in Singapore. Places that are more known among the GBMSM community may include bars or clubs that cater to this specific community. Due to restrictive measures, they would not be able to meet up with their friends, who form the crucial part of their psychosocial support systems, thus leading to poorer mental health. Social support from friends plays a crucial role in maintaining mental well-being and this is especially applicable to sexual minorities who are subjected to stigmatization (Hawthorne et al., 2020; Weinstein et al., 2017). Hence, the impact of COVID-19 on GBMSM will be considerably intensified, compared to their counterparts (Bishop, 2020).

Communities of GBMSM also face heightened vulnerabilities in the context of HIV and other sexually transmitted infections (STIs) and face more barriers in seeking for healthcare services (CDC, 2020). They may feel hesitant to seek medical support due to fear of rejection, resulting from society’s prejudice attitudes (Jackson et al., 2016; Shetty et al., 2016). These risks for HIV and STIs have been exacerbated by COVID-19, with more GBMSM reinitiating sexual activities once restrictive measures were relaxed, to compensate for their lack of social interaction during the CB period (de Sousa et al., 2021). With restrictions on non-essential health services such as health screening and counselling during the CB period and the gradual re-opening phases, several organizations that provide pre-exposure prophylaxis (PrEP) or post-exposure prophylaxis (PEP, and free screening for GBMSM were not able to continue operations. Hence, GBMSM were denied their supply of PrEP or routine screenings, which may contribute to the spread or delayed detection of STIs or HIV. GBMSM who were undergoing counselling therapy were denied their usual in-person therapy sessions due to the restrictive measures. These were detrimental to their health outcomes as treatment was delayed.

Singapore is still a conservative society that does not have existing policies to protect sexual minorities. A recent study conducted via a world-wide gay social-networking application reported that GBMSM experienced a huge impact on their mental health and faced more difficulties to access the needed health services during COVID-19, which highlighted the social risk factors and health disparities that existed prior to the pandemic (Santos et al., 2021). Especially in times of an unprecedented event (in this context is COVID-19), GBMSM will face even more stress with the lack of specialized health services catering to them (Arreola et al., 2015). The annual PinkDot event held in Singapore, which supports and spreads awareness for the lesbian, gay, bisexual, transgender and queer (LGBTQ) community, reported an increase in the number of attendees over the years (The Straits Times, 2018). This shows that more people are aware of the community and are showing greater acceptance of them.

As a conservative society like Singapore, such initiatives are insufficient to shed light on their experiences. Hence, it is crucial to understand how a pandemic like COVID-19 can impact GBMSM in the aspects of sexual and mental health, together with substance (including alcohol) usage (Sun et al., 2020). This can bring light and better engage appropriate stakeholders to develop interventions or policies to address these concerns. These findings remain relevant in a post-COVID-19 era, given that they highlight fault lines that drive health disparities for GBMSM in Singapore that have been exacerbated by COVID-19.

The primary aim was to explore the impact of COVID-19 on GBMSM in aspects of sexual health, mental health, substance use and access to health services in Singapore. The research questions were: (i) during the COVID-19 pandemic, what were the factors that affected their mental and sexual health; (ii) what were the possible barriers that impact their health-seeking behaviour during this period; and (iii) how could policies/services be enhanced to address these issues faced by GBMSM in Singapore during unprecedented times?

## Methodology

### Study design

An exploratory qualitative method with semi-structured interviews was adopted for this study.

### Recruitment method and participants

Self-identified GBMSM were recruited via purposive sampling, through posting study recruitment messages on the social media platform Facebook. Purposive sampling allows researchers to clearly identify the population of interest and ensured diversity in participant demographics and behavioural attributes (Palinkas et al., 2015). The study team recruited participants of varying demographic attributes, relationship statuses, as well as HIV or STI risk-related behaviours such as levels of sexual activity or substance use. These criteria were stated in the recruitment messages posted on social media, in order to ensure that participants recruited possessed characteristics that the study required. Contact details of the study team were provided in the recruitment messages. When interested participants contacted the team, the study team members assessed their suitability before explaining the study details and officially recruiting them into the study.

Sixteen interviewees were recruited for this study. The majority of the participants were of Chinese ethnicity; identified as gay, with one who identified himself as bisexual and another who identified as queer. Seven of them reported experiences of substance use and six of them were in romantic relationships. The interviewees’ demographics can be seen in Table I.

**Table I.** Demographics of participants.

Index	Age	Race	Sexual Orientation	Had ever used substances (Yes/No)	Relationship Status
IDI001	29	Indian	Gay	Yes	Single
IDI002	42	Chinese	Gay	No	Attached
IDI003	25	Burmese	Queer	No	Single
IDI004	25	Eurasian	Gay	No	Attached
IDI005	30	Chinese	Gay	No	Single
IDI006	28	Chinese	Gay	No	Single
IDI007	21	Chinese	Bisexual	Yes	Single
IDI008	27	Bangladeshi	Gay	No	Single
IDI009	34	Chinese	Gay	No	Attached
IDI010	31	Chinese	Gay	No	Attached
IDI011	34	Chinese	Gay	Yes	Single
IDI012	34	Chinese	Gay	Yes	Attached
IDI013	24	Chinese	Gay	Yes	Single
IDI014	30	Malay	Gay	Yes	Single
IDI015	24	Chinese	Gay	Yes	Attached
IDI016	44	Chinese	Gay	No	Single

#### Eligibility criteria

Inclusion criteria included participants who were aged 18 years and above and self-identified as GBMSM. It is crucial to understand experiences of citizens, PRs and foreigners as foreigners might have differing views towards the GBMSM situation in Singapore as compared to the other two groups of GBMSM. GBMSM across various nations encounter distinct sociocultural, legal and political landscapes. Comprehending these differences enhances our understanding of the influence of these elements on health, wellbeing and social interactions (Wickramage et al., 2018).

### Data collection

Semi-structured individual interviews (IDIs) were conducted for data collection, from January 2021 to May 2021. Semi-structured IDIs with an interview guide enable participants to express themselves freely, thus generating quality in-depth responses (DeJonckheere & Vaughn, 2019). Prompts and probes were used to encourage participants to provide in-depth explanations, thus generating more comprehensive data (DeJonckheere & Vaughn, 2019).

An interview guide with open-ended questions was developed from the study’s objectives, with prompts used to generate more in-depth responses (Doody & Noonan, 2013) (refer to Appendix). A pilot test was conducted on a self-identified cisgender gay male by one of the researchers, XY (a heterosexual female), to review the flow of the interview and ensure the clarity and relevance of the questions to the study.

All IDIs were conducted in English Language, via online conferencing platforms (e.g., Zoom) based on participants’ preferences. Specific weblinks were provided, ensuring only involved participants could attend the respective sessions. The participant information sheet and interview guide (see Appendix) were sent to potential participants prior to interview sessions, so that they could review the questions. Verbal consent was obtained before each interview commenced. Participants were told in advance not to mention names of substances they consume and had the option to switch on or off their video cameras. This ensured that all participants felt comfortable and safe to share freely. Two of the researchers, RT (the PI and a cisgender, gay man) and XY conducted the first two interviews together, and the other 14 were conducted by XY. All interviews were audio-taped and transcribed verbatim. Interviews lasted between 40 to 90 minutes. Recruitment ended after data reached saturation and no new theme was produced after discussion among the study team (Saunders et al., 2018). Each participant was reimbursed with SGD50.00 cash for their participation.

### Data analysis

The framework method was adopted for data analysis, as it is flexible and applicable for inductive or deductive or combined approaches. It allows study team members to have a clear overview of the coding frame during analysis process (Gale et al., 2013). There are seven stages in the framework method of analysis (Gale et al., 2013): (i) transcribing verbatim of all audio-taped interviews; study team member XY and MY transcribed all interviews; (ii) being acquainted with data by revisiting transcripts or audiotapes (cross-checking of transcripts was done by study team members after each transcript was completed); (iii) coding of data: for this study, analysis of the initial few transcripts was carried out using a deductive approach, utilizing the interview guide to come up with initial codes. Two members (XY and RT) coded the first two transcripts independently using the deductive approach, before coming together to discuss the codes, and came up with the initial coding frame; (iv) generation of a framework for analysis: the study team gathered to discuss the codes after analysis was completed for the first few transcripts, to form a coding frame for analysis of other transcripts; (v) analysis was continued with the existing coding framework and NVivo (released in March 2020) software (QSR International Pte Ltd, 2020) was used to keep track of existing codes so that they are accessible for subsequent analysis work. The study team members met iteratively to discuss the codes; (vi) the study team arranged codes into the framework matrix and tagged relevant quotations to codes; and, lastly, (vii) data interpretation; whereby study team members scrutinized the data to discern patterns, linkages and justifications, before coming to a consensus for the final three themes and seven sub-themes.

#### Rigour of data

Credibility is one criterion in ensuring rigour, which refers to how well findings represent the original views of participants (Singh et al., 2021). To strengthen credibility, probing as a type of iterative questioning was used in this study (McGrath et al., 2019). This approach allows for the discovery of subtle insights and ensures comprehensive data gathering. The study team utilized prompts and open-ended questions to clarify unclear responses and delve deeper into participants’ insights to draw out detailed and rich information, thereby enhancing the validity of the research findings.

Reflexivity in the form of a diary was kept throughout the data collection process, to keep track of preconceived ideas that may influence data analysis. It also enables researchers to recognize their unconscious bias. This ensures trustworthiness of findings (Teh & Lek, 2018). Study team members involved in data analysis kept a diary each and reflected on their preconceived bias prior to analysis and reflected on them, to ensure that their personal experiences did not influence the analysis of transcripts. Peer debriefing was conducted when codes and themes were discussed and finalized by three study team members (RT, XY and XM) of different sexual orientations: a heterosexual female, a cisgender gay male and a heterosexual male. This ensured no preconceived bias and personal experiences were in play, as the team members were able to highlight to one another if any potential bias was present during the analysis process

Transferability which looks at whether study findings are generalizable in different settings, was ensured, with the study team providing detailed elaboration of participants’ demographics and the data collection process (Singh et al., 2021). Data saturation was also achieved to ensure transferability of findings.

Dependability refers to the duplicability of the study to derive comparable findings (Singh et al., 2021). The study team provided an extensive description of study procedures so that readers have a clear picture of the processes and are able to replicate this study, thus enhancing dependability (Singh et al., 2021).

Confirmability pertains to the extent to which the study’s findings, interpretations and conclusions are objective and impartial, grounded in the gathered data rather than influenced by the researcher’s own beliefs or preconceived notions. Through multiple peer debriefing during the study process, the team ensured that analysis was grounded to data instead of personal beliefs (Maher et al., 2018).

### Ethical considerations

This study was approved by the National University of Singapore Institutional Review Board (Ref: NUS-IRB-2020-58) prior to the commencement of the study.

## Results

**Figure 1.**
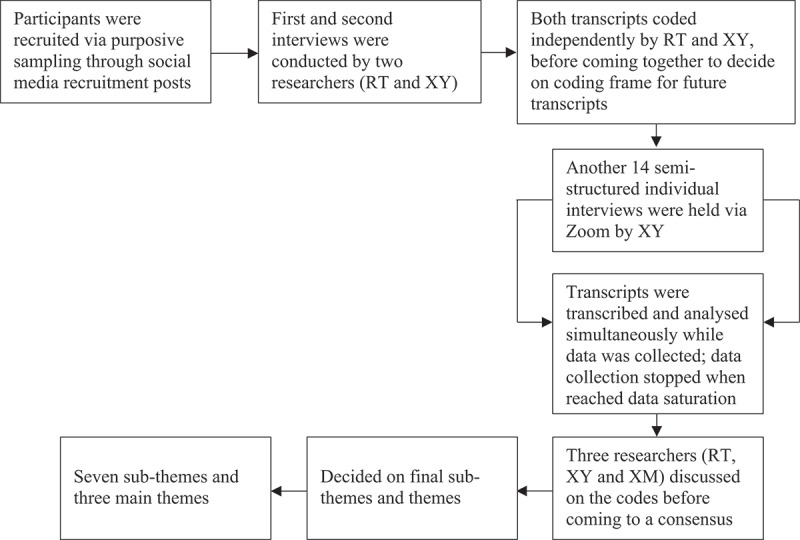
Study flow diagram.

Three main themes and seven sub-themes were generated (see Table II). The first and second theme focused on mental and sexual health of the interviewees, while the third theme explored GBMSM’s access to health services during COVID-19. A study flow diagram (Figure 1) gives a summary of the study process.

**Table II.** Themes and sub-themes.

THEMES	SUB-THEMES
COVID-19 & Mental Health	Increasing negative sentiments
	Changes in coping mechanisms during CB
	Experiences with “staying-in” during CB
	Impact on dynamics of romantic relationships
	Positive experiences during a pandemic
COVID-19 & Sexual Health	Changes in personal sexual beliefs
	Impact on sexual activities
COVID-19 & Access to health services	

### COVID-19 and mental health

This theme has five sub-themes, which explored interviewees’ personal experiences with COVID-19, emotions felt and how they coped with the ever-changing regulations during this period of uncertainty. The impact on relationships with families and partners will also be discussed.

#### Increasing negative sentiments

The majority of the interviewees felt negative emotions during the pandemic, with CB being the most impactful phase. During CB, physical social interactions beyond households were limited to almost zero, which led to many interviewees feeling lonely and depressed. This sense of loneliness was especially amplified among GBMSM who did not open up to their families with regard to their sexual orientation. Due to the restrictions imposed, entertainment centres that cater to GBMSM were also temporarily closed, resulting in them “losing” a form of support in their lives as these places were considered safe sanctuaries for them to be themselves.

Initially, it was really hard to bear it. Especially because you really couldn’t see anyone, there was no excuse for social gatherings … I had to occupy myself, go for a run sometimes, going grocery shopping, and even cooking … I did so many new things like out of desperation because I really couldn’t stand being cooped up at home for so long … you just crave that that human presence … (IDI005)

But COVID-19 really has brought a lot of issues of loneliness to the surface, like you could you could do a lot of you could do a lot more things before in the past uncover hidden, like you hide the loneliness that you feel whether you’re gay or not, but perhaps more prevalent to gay men. And then I think COVID-19 really prevented people from continuing continuously sweeping things under the carpet that would be my, my, er I suppose overall view of that. … I think it just build, they build on each other. … circuit breaker was probably terrible. I really don’t think life is going to go back to normal to pre pandemic days. So yeah, so so it hits LGBTQ people harder and gay because a lot of them lead double lives. who I am at work is not really who I am or who I am at home. Also, I won’t tell my parents lot things. Or when when I’m with my friends, I really can say the things that I want to say but I cannot say I think I think for closeted people it’s really a double life, you and it could be it could be like you are a spy, you are a spy, you have to say you cannot say the things you want to say because it will reveal who you are. (IDI002)

What COVID did to like, gay and bisexual men, … they exacerbated a lot of the existing problems, because people can go out people can go to their safe place or spaces. Like the first place that I went into … the first bar I went to when dining in was allowed in restaurants and bars, was [gay bar]. This is like, I felt like that place was home since 2016. So that was the first place I rushed through. And you couldn’t do that during circuit breaker, you can’t do that now. So that’s why I feel like that’s the kind of like situation that I face um that a lot of gays face … I’m still really bummed about the fact that there’s no PinkDot for the second year in a row in person. Um, you know, PinkDot, honestly, once a year, I always look forward to it, because it’s the largest public display of like, an acceptance for the LGBT community that I always see., I will get in my head a lot, you know, about like, all, you know, I think if there was no PinkDot, it would get in my head a lot. Like do people hate us? Yeah, there has taken steps to make it virtual and stuff, but it’s just not the same. And it doesn’t feel as much of a celebration, and I’m quite bummed that this year we won’t get it again. (IDI015)

Due to the evolving COVID-19 situation, there were a lot of uncertainties regarding restrictions imposed and some expressed anguish and hopelessness towards the situation.

Occasionally, I would look out and see the roads outside my house, basically like very dead, like a ghost town … see every single person with masks on … even till now sometimes, that sight looks a little like dystopian to me … I know, like, how far reaching and how devastating the situation is across the world, and how our lives have been irrevocably changed by this pandemic, like sometimes I feel it’d be a bit of chilling despair … (IDI005)

Besides having to deal with new regulations, some interviewees started viewing themselves negatively due to the stress which stemmed from CB and other external factors like the inability to maintain personal financial stability.

I mean like one random like observation that I had was that I was considering consuming a lot more social media during the circuit breaker period because there’s nothing much else to do. And I think that consuming social media, let me to like looking at the lives of other people, like, it’s like, all the different activities I know, they usually go to, or even like how they are spending their, like, circuit breaker time doing something productive, or they know what during that home workouts and showing their bodies, and I think all that like, definitely made me like more like, made me more envious. Yeah. And like, maybe like made me more unhappy about myself, my own my own body, because I notice that all these other people that are rocking, like you know the circuit breaker period whereas like, I’m just like, barely getting by. So there was like jealousy. And yeah, so I think that circuit breaker really did like make me less healthy, like both mentally and physically. (IDI001)

I couldn’t find jobs, because a lot of companies they were definitely affected by this … it’s probably quite hard to find a job even if I want so definitely stressed because I rent the room by myself … I still have to pay for my therapy sessions. (IDI006)

#### Changes in coping mechanisms during CB

Due to the emotional changes that they experienced, especially for those who were substance users, the psychosocial impact of COVID-19 on their mental well-being was more significant. Interviewees who were substance users reported increased dependence or even the dosage on substances during CB to alleviate their stress and emotional burden stemmed from loneliness or stress from family

… because of the lockdown, we are all in our own rooms most of the time and we are all by ourselves … we tend to use drugs more … typically we could be outside doing healthy stuff but now that we are cooped stresses us, it also amplifies our need for the form of substance … because when we go out right it’s not just about going out it’s also for us to relieve stress. It’s like a coping mechanism sometimes it’s a way to de-stress, so that is taken away from us … taking drugs becomes like the next in line. It’s some medication in a sense. (IDI011)

Because, compared to last time when I meet my partner right, we will take together for that session. But because circuit breaker like everybody is close and yea, you know, how this … we have more of a personal time a very me-time you see, so when during the free time got nothing to do, right? You don’t know what to do, right? So, we tend to actually take more substance … to alleviate some sort of stress … I don’t feel happy in fact I feel very stressful. So I’m actually quite reliant on the substance itself … family issue as in day in day out, you will see your parents or even probably your siblings, then they’re bound to have some sort of like they call it drama whereby your family will argue with you, to talk to you because of the time spent together is really too much already … the dosage is getting higher. (IDI012)

Besides using substances as a coping method due to the isolation brought about by COVID-19, a few interviewees shared the loss of their usual coping mechanisms during pre-pandemic days. Some mentioned that being in gyms was a form of coping mechanism during their stressful periods, while some shared that being able to converse with their partners in person was their method of releasing their stress.

Uh okay as superficial as it sounds I think because most of us we actually live in the gym … the lockdown affected us and it’s not just a physical thing, at least for me it is a very mental thing, like I take a mental break from life … with that I lost the avenue. (IDI011)

Yeah, I mean because the person that I knew who will support me is my partner, so like that was the person I wanted to talk to, I will like I need to tell you about my day, I need to tell you how stressful it’s been like I feel so bad for my colleagues but that person wasn’t there. There are other avenues or like sources that I can seek help from but I wanted it to be my partner because they know what’s going on. I didn’t want to start from scratch and be like okay, this is what has been going on y’know is because this is an ongoing theme right, but I couldn’t talk to them, they weren’t being there for me … so all of it was just building up. Ya, so it was like a little- like a small little atomic bomb that was waiting to explode, so I was like holding all these things in, I was holding all these things in. I lost it at work, so I got angry on my colleagues. (IDI001)

#### Experiences with “staying-in” during CB

Apart from intrapersonal issues faced, CB required everyone to stay at home for majority of their time which led to a lot of frustration and strained relationships with family members. Not all participants were out to their families regarding their sexual orientation, thus some shared difficulties faced due to increased interaction at home. They had to be more self-aware or “modified” their behaviours, in fear of exposing their sexual orientation.

It was quite bad, I couldn’t go out and meet my friends … going to a bar or going out to clubs and meeting my friends is usually I feel like where I can be like myself … being stuck at home was very hard, because I’m always under the impression that my parents are homophobic. I just felt very, under surveillance, almost like I cannot really be like myself and had to be on “tip-toe” because everyone is at home all the time. That wasn’t very pleasant. (IDI015)

I will be very transparent with you, I told you earlier that I didn’t like to stay at home. It’s because my relationship with my family it’s not very good, because I’m not straight. Uh ya our relationship is not very good because I came up to them when, when I was in poly [polytechnic]. They told me a bunch of things like that I will never be successful. If I don’t end up with a girl, then I’m going to fail and talents or whatever. … My when they asked me like, why don’t I just choose girl, I was also a bit stunned … I can’t give them an answer because I am attracted to guys. Then with that, it has just been really bad. … During the circuit breaker I was forced to stay at home and it was honestly the first time I had to really live within the same space for so long. I mean, I grew up in my house, but I never had to see them for so long. … Yeah, at the start it really made me feel very anxious. Because I didn’t know what I was going to do. So most of the time, I just keep in the room. And honestly, sometimes now I still do … I just don’t like talking to them. … It’s very tiring so I just hide away you know. Within the same household, but I still hide away. So more like, we eat the exact same thing, just that within the same house but I am just not with them directly by the side. (IDI007)

#### Impact on dynamics of romantic relationships

Interviewees in relationships were greatly affected by the restrictions due to the reduced physical time they had with their partners, as most do not live together. The majority mentioned that sex played an important role in relationships of GBMSM and the lack of physical interaction led to poorer sexual relationships which was a commonly faced issue among participants.

We cannot interact in any other ways other than online, so I think it has been limited in a lot of ways. We don’t see each other as much face to face in person, our text communications also became more virtual … I sort of don’t remember him after a while … for us gay guys [who] use drugs for sex, because of COVID we are not able to meet up there’s a lot of restrictions, we are only using it at our own end and that really impacted our relationship. (IDI011)

I think sex is a lot of a higher importance in any gay relationship. Um so definitely … one of the main features of circuit breaker and COVID is to reduce physical contact and I think definitely the impact on the gay couple would be a lot worse. I heard a lot during circuit breaker I know of at least four gay couples who broke up. Um but I didn’t hear of any straight couples but then again my friends by this age are probably married and so they don’t break up as easily as the gay people so my feeling is definitely the physical, um the strains of the relationship is definitely higher from the gay couple without sex and because if you take sex away from [us], usually between the two gay guys, there are a lot of things that conflict, two people if they have different, if they different sexual appetite, so that even with or without COVID can cause a relationship problem already, one person wants sex then the other one doesn’t want as much … But now you add in one more layer of law what if the person who wants sex have a certain disregard for law [lockdown-related laws that prohibit individuals from visiting other households], the other person might want sex equally but if the person is a bit more by the book and didn’t- doesn’t want to break law for sex and that could be another cause of conflict as well. … That’s the kind of mind-set I think the gay community have for sex, like um you cannot have a sexless relationship. (IDI009)

However, one participant mentioned that his relationship with his partner improved as they had more time for each other during CB, as compared to pre-COVID days as they were all stuck at home due to the restrictions.

COVID-19 did strengthen our relationship, emotionally, because we actually communicated a bit more frequently over call … maybe an hour every night or so just talking to each other catching each other up. (IDI004)

#### Positive experiences during a pandemic

It is evident that COVID-19 brought about inconveniences and new practices that impacted significantly on individuals’ mental well-being, but some participants actually highlighted certain aspects of their lives that were improved. Some reconnected with old friends via online platforms, which improved their social lives. Some mentioned that COVID-19 brought about self-improvement by providing the space that they did not have previously, to self-internalize and address issues themselves instead of depending on others.

Because of circuit breaker there were a lot more soul searching, you have to do a lot more of the situation analysis within myself because I don’t have anybody easily to bounce ideas off so I found myself dealing through a lot of my own problems which I never, which in the past I could just vent it on others … I realised like okay … and self-reflect. … I felt like oh dear, I need to work out things by myself and I found myself doing um going online, going Google to- to look for relationship advice which never had to do in the past. So that- that was a- that was a new experience but um, was that definitely- was- was it a bad thing? I don’t think so, I think it also make me a bit more um, uh reflective and that I could process them to like it’s a bit silly to say this at 34 years old but I tend to become a bit more adult and process my problems first before just ranting about it. (IDI009)

Some interviewees who previously took alcohol in high amounts due to regular social events also experienced a substantial reduction in alcohol consumption during and even after the CB period.

I will say I cut down a lot during after COVID yeah because cannot go out and like, then cannot drink only can drink at home … I will say a healthier amount now. (IDI008)

I think I’m quite a social drinker last time, but I think the bars are closed, right? The good thing is that, like, I become a lot less alcohol dependent, dependent for a lack of better word. Uh so that’s a good thing. (IDI003)

As for those substance users, it was shared that there could be a decrease in substance consumption due to tightened supply caused by border restrictions. This had resulted in a price surge for substances and users might not be able to afford them.

… during circuit breaker, substance actually surge a very ridiculous pricing. So because of the surge of the pricing, I think most of the substance user, they also tend to give up a substance already … they use less. (IDI012)

However, a deviant case shared that, with a price surge for substances, those who were still taking substances were portraying their social statuses instead of using it as a coping method.

Because of prestige … I have the power and money to gain access to some of the chemicals that you guys cannot get it. I have that kind of connections to this. You all did not. I have ever asked some of them before. And these are the people who are from the elite circles. (IDI016)

### COVID-19 and sexual health

#### Changes in personal sexual beliefs

COVID-19 not only significantly affected the mental health of GBMSM, but also contributed to changes in their sexual beliefs. Due to restrictive measures, men were unable to have sexual relationships with others. This was important as many felt that sexual relations were an important part of building intimacy and connection within the GBMSM community. This triggered some to develop stronger desires towards sex and engage in sexual activities more actively once regulations were more relaxed.

Definitely the pandemic has affected [me] in some ways that, after going through that one round of two months of no contact I felt how need-deprived I was … I feel like when these restrictions are lifted, I have to like, spend all the sexual currency that I have … (IDI015)

I don’t know about the heterosexual community, but … it feels like hooking up [in the GBMSM community] is a norm … I think quite sad because when the restrictions came about there was just too many stories found on the papers of people getting caught because they were visiting people … for various reasons and I think a lot of people commenting that obvious reasons is to hook-up because you’re saying this guy is that at another guy’s place … which of course even if you’re best friends you wouldn’t take the risk right of going all the way there to meet your friend and all that … so it was a little bit scary. Because I mean there also stories of heterosexual people, heterosexual couples taking the risk to visit each other but you don’t see that many as compared to those getting caught in the gay community. (IDI013)

Despite developing strong sexual urges to satisfy, the majority shared no significant change in their safe sex practices, which included usage of condoms or PrEP. However, some reported having “additional” safe sex practices, which included “screening” sex partners for the possibility of exposure to COVID-19 due to their fear of acquiring the virus.

… it did shape the way I feel about hook-ups and safe sex, it meant more than just condoms and PrEP and probably now you talking about whether you should have mask or respiratory protection … So sexually transmitted COVID was a fear as well … if a person has COVID, the transmission during sex is definitely positive and COVID really change my sexual attitude. (IDI009)

#### Impact on sexual activities

During certain phases of COVID-19, places like hotels or saunas were closed, thus there was no appropriate venues for sexual activities. Participants voiced their fear of getting apprehended for going against regulations or meeting others for sex. Therefore, the majority shared a decline in sexual encounters.

I was very paranoid about getting caught and fined … I’m not going to risk going to jail getting fine and getting COVID touchwood, like for a one-night thing … (IDI008)

I stop going … you can imagine [for] gay couples to have a physical location sometimes can be difficult for sex and therefore, with my partner sometimes we used to go to saunas just because there is a possibility of a place … and hotels. But since COVID then that behaviour stopped because I also don’t want sia suey [“to be embarrassed” in Hokkein dialect]. [If I get subject to] contact tracing then this whole sauna thing also appear [in my past visited places]. I think saunas and hook ups definitely stopped because I really didn’t want to [get contact traced] to those people. (IDI009)

However, some shared their inability to control sexual urges attributed by being cooped up at home. They engaged in riskier sexual behaviours like meeting random people from online platforms who were seeking a sexual rendezvous, to have sex in public areas which could have gotten them exposed to not just STIs but COVID-19.

I actually did change my sexual behaviour … because I cannot bring anyone home because my parents are home. I had sex at the staircase. And previously, I was not very keen on public sex … that was during circuit breaker last year … I think that was during circuit breaker last year, um, and I think what triggered me to do it was because I think I think during circuit breaker, on Twitter, like some like anonymous porn accounts were like, hooking up at Macritchie Reservoir. I tried things like public sex and everything, which I don’t usually like to do. (IDI015)

Many also adopted alternative methods for sexual activities, with the majority highlighting the usage of online platforms.

I mean, sounds a bit ridiculous to say but virtual hook-up … nowadays technology is very advanced, one of the sex toys I have is a vibrator which can be controlled with the internet. So that was one way that I kept things alive for myself with cyber-sex. (IDI0015)

I feel for me I wasn’t having physical sex *per se* but I mean I made it up, we had other avenues, uh yeah so we’ll just turn to like the internet, so it will be like a lot of reliance on pornography, that’s one thing … also sometimes like uh, we would just like video call each other and try and like have Camsex [online camera-mediated sexual activities] then we’ll just do that and I think yeah that was quite fun also, I think that kind of kept us quite good … we found our way around it so like we’ll set aside the time like maybe twice a week where we’ll just like video each other and relieve ourselves. (IDI001)

### COVID-19 and access to health services

In addition to the impact of COVID-19 on mental and sexual health, operations of health services that cater to these two areas were also affected. They were considered non-essential and had to cease operations temporarily. Some participants brought up issues of not being able to get appointments for both mental and sexual health services.

… possibly affected me the most was in terms of counselling services … mental health services … they were not considered essential. I started seeing my therapist maybe just before the restrictions kicked in, I saw him like twice so when the restrictions kicked in obviously, we can’t meet face to face anymore. … it really affected me because it will help a lot to know face-to-face with a human … That was something that really irked me a lot. (IDI006)

I was supposed to go for the [community-based organisation] free [HIV] screening and it’s all cancelled … So I am slightly annoyed. I did also want to get my PrEP prescription … (IDI015)

To be honest with you, I try to like I tried to go for testing during pandemic, I think the clinic was closed or something or like the appointment. Like on the website, to do some appointments or something like that. And I couldn’t like do the appointment thing, or somehow it’s not as convenient anymore. I think ya I wanted to go for testing. I think during during the circuit breaker … I think the [sexual health clinic] I think I couldn’t navigate calendar system or something like that. Because I think there were many information about restrictions. I also didn’t know if it was closed or open. I tried, I tried going but I tried accessing but then I was like nope. But at the same time I was also seeking counselling, [community-based organisation] is it? Yeah. I was I was trying to book appointment but then that one also very complicated. The website reminded me more about COVID than the services they providing, so I just give up. (IDI003)

Due to these disruptions and COVID-19’s impact on one’s mental well-being, participants brought up some suggestions that could possibly ease the brunt of the pandemic. For GBMSM who need mental support but may not be out to their families, having to use teleconferencing platforms for counselling may not be feasible. Participants shared some alternative methods that can address this issue.

… during circuit breaker for people who like, who can’t be themselves or can’t talk about it, because their family are around right? But they need to talk about it and they want to be heard. So texting will help, you know, I mean, like, yeah, so at least they still can let out to a professional … without like, “exposing themselves” or coming up accidentally to family members. (IDI008)

Some mentioned that social support groups are available for GBMSM, but might not be known to all, so raising public awareness towards these resources is critical.

I think it’s important that these resources have to be publicly publicized so like is more known to a lot of people because when I told my friends like [community-based organisation] and then they’re like what’s [community-based organisation]? I told them it’s like a LGBT counsel service, have you not heard of it? They’re like no, I only know [community-based organisation] and that’s it … (IDI001)

The point on having mental and sexual health categorized as essential services was also discussed. Those who were actively seeking mental health support or required sexual health services were denied the access to these services during the CB period.

I think that it was quite tragic and devastating that a lot of people who needed therapy were denied, that’s very important for them. I personally felt that during that period, the rules could have been relaxed. So I do strongly feel that this is something that should be made available to them, especially knowing like the pandemic like takes on healthy, normal functioning people, much less known than people who are struggling with mental issues. (IDI005)

## Discussion

Sixteen self-identified GBMSM were interviewed with regards to COVID-19 and its impact on their mental and sexual health. They shared experiences on how COVID-19 influenced their sexual lives and its impact on intrapersonal and interpersonal aspects of their lives. Issues faced with access to mental and sexual health services throughout the pandemic situation were also discussed.

**Figure 2.**
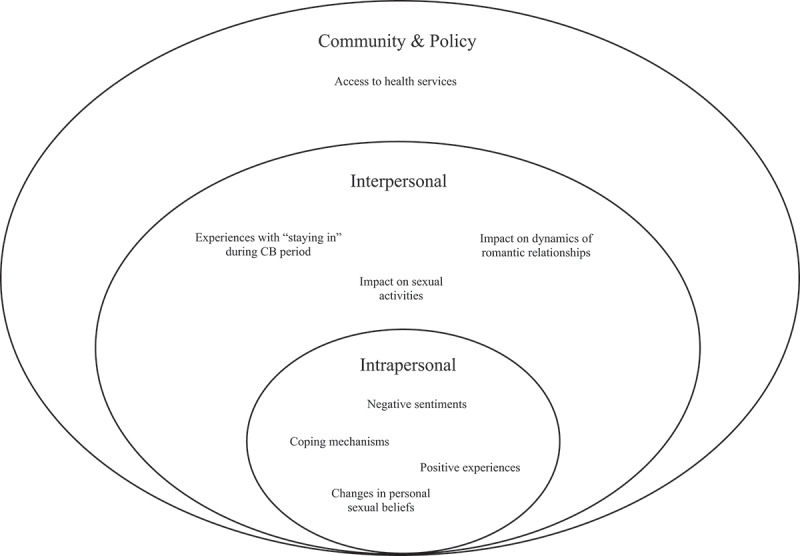
Socio-ecological model of COVID-19’s impact on mental and sexual health of GBQMSM.

The results gathered from the interviews will be discussed at the respective levels of the SEM (see Figure 2). The SEM enables better understanding of individuals and their affiliations with social connections and community at large (McLeroy et al., 1988). Based on the range of issues that participants highlighted in the interviews, we sought to use the SEM to frame our discussion as it can elucidate the multifaceted experiences faced by GBMSM during COVID-19. This will be discussed through the four different levels of the SEM: intrapersonal, interpersonal, community and policy.

### Intrapersonal level

At this level of the SEM, we looked at how negative emotions experienced by participants affected their mental and sexual health, thereby influencing their behaviours towards sex and substance use. The majority of the participants shared negative sentiments such as feelings of loneliness, and negative perceptions of themselves during COVID-19 (see Figure 2). Studies showed that GBMSM seek support and comfort mostly from the GBMSM community (Liu & Lu, 2019; Schmidt et al., 2020), due to the existing discrimination they face in society. A survey conducted on a global scale reported that GBMSM experienced a significant level of mental burden such as stress and loneliness during shelter-in-place measures (e.g., CB period) (Greenhalgh, 2020).

#### Change in behaviour towards substance use

Participants who were substance users reported increased substance usage, specifically during CB when more intensive social distancing measures were imposed. In unprecedented situations like COVID-19, psychosocial connections will be weakened, building on the existing susceptibility of GBMSM towards mental health issues. This may result in them turning to alternative coping methods which are usually maladaptive behaviours, especially for substance users. Studies had reported that substance use among GBMSM were related to poor psychosocial well-being, such as being depressed or anxious (Evers et al., 2020; Prestage et al., 2018). The American Addiction Centers (2019) commented that dependence on substances is a common coping mechanism for those facing psychosocial issues. In this context, participants reported negative emotions in relation to COVID-19 and its restrictive measures, thus an increase in substance usage reflected their coping mechanism.

#### Change in sexual behaviour

Besides affecting their mental health, COVID-19 also had a significant influence on their sexual behaviours. Due to restrictive measures, participants were devoid of sexual contact with others, thus causing a build-up of sexual frustration. Some developed stronger inclination to engage in sexual activities, even if previously they might not believe in having casual sex. Being in a frustrated or restricted state of mind could have led them to change their behaviours towards sex. This was also reported in another study that, due to restrictive measures, casual sex among GBMSM increased (Sanchez et al., 2020). However, their beliefs towards safe sex practices were not affected, with the majority continuing practise what they did prior to COVID-19. An “enhancement” to existing safe sex practices was mentioned by some, which was being more aware about possible exposures to COVID-19 of their sex partners. This could be due to their fear and uncertainty towards the pandemic as cases in Singapore were ascending rapidly during that period and no vaccination was available then. A study reported GBMSM modifying their usual sexual practices such as reducing oral contact with their partners, in fear of contracting COVID-19 through salivary secretions (Shilo & Mor, 2020).

#### Positive influences on self

Some participants had positive experiences in the face of COVID-19, essentially the point on having time to rediscover and improve themselves. Being restricted to their own homes during the CB period, participants shared about experiencing individual growth and being able to face problems and regulate themselves more independently, as compared to pre-COVID days. Emotional resilience is one aspect that will be challenged during a crisis and being able to get something good out of this situation reflected how it helped these participants strengthentheir emotional resilience (Fioretti et al., 2020). A study by Yu et al. (2022) highlighted that participants reported experiencing individual growth during the peak of the COVID-19 period. In the face of an unprecedented event, individuals discovered how to regulate their own emotions and explored alternative methods of support which includes self-reliance. Hence, individuals would feel a sense of growth especially in their level of resilience.

### Interpersonal level

At this level, we looked at how social, family and occupational relationships affect individuals’ attitudes and behaviours. Negative relationships were seen to exacerbate mental health issues, thereby contributing to undesirable health outcomes.

#### GBMSM and family

Due to restrictive measures in place, participants were placed at a higher risk of accidental disclosure of sexual identities to family, thus they had to modify their behaviours to adapt. Participants had to be aware of their usual behaviours at home and more conflicts with family members were faced during this pandemic. The inability to be themselves is a common issue faced among GBMSM, especially those whose sexual orientation is not disclosed to their families or friends due to societal stigma (Conley et al., 2018) (see Figure 2). This will further contribute to their failing mental health as they faced daily stressors of being at home and under the scrutiny of family members on their behaviours.

#### GBMSM and their partners

The lack of physical closeness with partners also led to the demise of romantic relationships among participants who were then attached. Many mentioned that not having physical sexual connections or face-to-face communication resulted in negative attitudes like mistrust and waning relationships. The majority of GBMSM do not reside in the same household in Singapore, as they are unable to purchase new public housing together. Under Singapore’s law, only married couples are able to purchase new flats. Singles have to wait till 35 years and above before they can purchase flats alone (Keating, 2018). Intimate relationships among GBMSM are considered crucial in maintaining health outcomes as partners serve as mental supports. Hence, during a situation like COVID-19, the inability to maintain physical interactions will contribute to strained romantic relationships leading to negative health effects (Li et al., 2020).

#### GBMSM and their community

During the CB period, for participants who were single, their usual sexual interactions such as group sex or other forms of physical sexual activities with others were shifted to virtual options via webcams or pornography, to satisfy sexual urges. The shift in sexual activities corresponded with the increase in negative emotions among GBMSM during COVID-19, as these activities allow them to satisfy their sexual drives and ease mental burden (Uzieblo & Prescott, 2020). However, some participants were driven by their strong sexual urges and need for physical gratification that virtual sources were insufficient and partaking in risky sexual activities like group sex became a norm, after restrictive measures were more relaxed. It is indisputable that COVID-19 had indeed influenced their patterns of sexual practices, due to the need for them to relieve the pent-up emotions accumulated during this period.

### Community and policy levels

At the community and public policy levels of SEM, existing relevant organizations, together with the governing body, have the responsibilities to hold with regards to the pandemic’s effects on people (McLeroy et al., 1988). Participants mentioned about being denied their usual counselling sessions and access to screening and PrEP prescriptions, which caused much frustrations and anxiety. The access to mental and sexual health services catered for GBMSM were impacted by restrictive measures imposed during CB period (see Figure 2). During CB, these services were defined non-essential as they do not contribute to the efforts towards controlling COVID-19. However, as evidenced by this study, the impact of COVID-19 on GBMSM is not minor. They are vulnerable to mental health related issues and it is critical that these services are made available for those in need, if not they might adopt maladaptive behaviours to address their issues.

### Strengths and limitations

One strength of the study is that two coders analysed the initial few transcripts to decide on the coding frame for future transcripts. The last few transcripts were also coded by two coders, ensuring that codes generated initially were still relevant and eventual themes were discussed and finalized as a team. This provided a deeper analysis to the data and ensured rigour (Church et al., 2019). Another strength is that purposive sampling was used for recruitment, thus participants with characteristics who fulfil study requirements were recruited. Purposive sampling also facilitates transferability of data from this study to future studies on participants with similar traits.

One limitation was that recruitment and data collection were conducted virtually. Potential individuals might not be able to join in the study due to the lack of access to the social media platforms used for recruitment. This would limit the types of participants recruited and the responses obtained. Participants might also be more distracted and experience tiredness more easily as compared to physical interviews (Epstein, 2020). This might result in responses that were not sufficiently in-depth. Another limitation is social desirability bias. As participants were asked to share their experiences with substance use, due to the sensitive nature of the content, responses provided might have been affected. However, prior to the interview sessions, participants were assured about the confidentiality of their data and were informed that no identifiers will be used. This would have encouraged them to share their thoughts freely. Points brought up by participants corresponded with studies which explored similar topics to this study, thus the study findings were seen to be good representations of participants’ experiences.

### Implications for public health and recommendations

The study findings highlighted how GBMSM felt stressed and lonely during the days of CB, how mental health support services such as counselling that provided minority communities such as GBMSM with the support for their mental well-being were halted, resulting in some of them adopting maladaptive coping mechanisms such as heavier substance abuse or riskier sexual behaviours. These findings enabled us to have a clearer idea on the situation to better engage appropriate stakeholders to develop interventions or policies to address issues faced during a pandemic.

At the intrapersonal level, more efforts should be placed on improving individuals’ personal coping skills and attitudes towards problems faced in life. Life-skill trainings like social-emotional learning (SEL) by agencies that provide services to GBMSM. This will enable GBMSM who are experiencing mental health issues, to self-regulate their emotions independently and cope with challenges they face daily. SEL can enhance their coping mechanisms, which is crucial considering the elevated incidence of mental health challenges this group encounters in comparison to their heterosexual peers (Mustanski et al., 2015). As a result, this can decrease dependence on substances as a means of coping. Additionally, SEL enhances interpersonal skills and encourages the formation of constructive relationships, essential for establishing supportive networks that are vital for recovery and lowering the likelihood of relapse from substance use. Hence, during a situation when professional services are not easily accessed, they will be able to make responsible decisions independently and be more mentally resilient. This will contribute to better mental health outcomes in the long run.

At the interpersonal level, efforts should be placed on training GBMSM to become volunteers with basic counselling skills and crisis management techniques, with focus on online services. With such peer-led support groups, GBMSM will feel less pressured and more comfortable to share their problems in a group of like-minded peers (Young et al., 2015). Mass activities online can also provide GBMSM with the platform to interact with others from the community, addressing the point on social isolation in the face of a pandemic. More online campaigns should be carried out to raise awareness regarding the presence of these groups, so that GBMSM know who they can turn to in times of need.

Campaigns can work with existing agencies (e.g., Out in SG) or applications (e.g., Grindr) that cater to the GBMSM community to promote their services and activities. Existing organizations that conduct outreaches to GBMSM community to promote safe sex practices and provide anonymous testing services (e.g., Action for Aids), should explore how to re-invent their services to make it align with measures implemented during a pandemic and the evolved situation during post-pandemic days. Perhaps health promotion efforts can be shifted online, making information more accessible. This will ensure a wider coverage without breaching regulations. Policies or governmental strategies like the inter-agency platform which was transformed from the COVID-19 mental health task force should put more focus on the mental well-being of minorities (e.g., sexual minorities), looking into how they can arrest early cases of mental illnesses and better provision of support help groups.

## Conclusion

This study highlighted the various effects COVID-19 had on the mental and sexual health of GBMSM, as well as its impact on access to health services. Participants mainly experienced negative emotions and experiences at an intrapersonal level and interpersonal level (with families or partners), which were also worsened by prevailing stigma that GBMSM already face in Singapore and within their social networks. Sexual behaviours associated with HIV and other STIs risk and substance use were seen to be maladaptive coping methods of social isolation due to COVID-19. These dynamics were all exacerbated by the closure of “non-essential” services, which included many important services for mental and sexual health that were relevant to the GBMSM community. Public health measures should explore easing barriers to health services for the GBMSM community, making mental and sexual health services as one of the priorities when planning service provision in times of a pandemic. Future interventional studies can examine the efficacy of online counselling services or online group activity sessions, on the psychosocial well-being of the GBMSM community and the efficacy of online health promotional efforts on safe sex practices, during unprecedented times of crisis.

## Appendix. Interview guide

**Table:**

Topics for discussion	Themes/Prompts	Guiding Questions
Introduction Demographics	Good morning/afternoon/evening, I am _______ and thank you for taking your time to join me in this online interview. This should not last more than one hour and the entire conversation will be audio-taped. This is to ensure that your responses are well captured. Some notes will also be taken down during the session. You can choose not to turn on the video camera if you feel more comfortable that way. However, no names or identifiers will be mentioned during the interview to ensure your privacy. All data collected will be kept confidential and only be shared among the study team. During the session, you can choose not to respond if the question or topic makes you feel uncomfortable. You can request to stop the interview at any point of time. We will be discussing some topics about the current global situation on Covid-19 and how it has impacted your daily living and activities, as well as your relationships and sexual health. Do you have any questions before we proceed?	
Experience towards Covid-19 situation	- Globally - Locally - Work (working from home? Alternate weeks of working from home?) - Social activities (gym/exercise classes at studios, gathering with friends/families or other leisure activities) - Schooling (if applicable: any changes to attending classes? Online? Self-directed learning?) - Any changes to daily transportation to and fro? - any changes to your living arrangement? (eg. had to shift to another house in order to care for parents etc)	How do you feel about the current Covid-19 situation? Can you share about your experience of the circuit breaker period? How did it affect your usual daily activities? How did a typical week look like for you pre-circuit breaker? How has it changed since the circuit breaker? (Can dive into differences across phase 1, 2, etc. of reopening)
Family dynamics Personal relationships	if did not mention about living arrangement previously Support from partner during circuit breaker period Family support towards sexual orientation/relationship	May I ask what your living arrangements are at this point? (with roommates, parents, alone, partner etc.) As this interview is exploring how Covid-19 has impacted gay or bisexual men in different aspects, may I know when did you start identifying yourself as gay or bisexual? Are you currently in a relationship? If yes: Is it an open or committed relationship?- Are you currently staying with your partner?- If staying apart: How did you manage your relationship during the circuit breaker period?- Were there any stressors that came up during this time? How did you deal or cope with them? Does your family know your sexual orientation? If yes, when did they know of it? How did they respond to it? If no, what are your concerns towards sharing your sexual orientation with them?- Has it been harder to keep your sexual orientation a secret from your family during the circuit breaker period?- Has your sexual orientation played any role in the dynamics you have with your family during the circuit breaker period?
Sexual behaviours	If interviewee has a partner- Perceptions towards safe sex practices (condom use?)- Perceptions towards risk of HIV or STIs? If interviewee does not have a partner - More engagement in virtual sexual services?- Engage in sex services? From which avenues (eg. spas? massage parlours?)- Perceptions towards safe sex practices (condom use?)- Perceptions towards risk of HIV or STIs?	As during the circuit breaker period, we were encouraged not to interact with anyone who are not within the same household, how did it affect your sexual relationship with your partner? What were your sexual behaviours like prior to Covid-19? What are the changes during this period (if any)? As during the circuit breaker period, we were encouraged not to interact with anyone who are not within the same household, how did it affect your sexual life? Prior to Covid-19, what were your patterns of sexual behaviours? During this period, what are some changes you have experienced towards your sexual practices?
Access/Utilisation of sexual health services	HIV or STIs testing, access to PrEP or PEP, management of STIs- Are you currently on any pre-exposure prophylaxis for HIV?- What adjustments have you made to your sexual life if you are unable to access to PrEP during this period?- Engage lesser in casual sex?- Increase usage of safe sex protection?	As we know that during this pandemic, there are many restrictions imposed on several areas such as entertainment areas and even health services. What are some of the barriers/difficulties you faced during this period when seeking for sexual health services?- Access to condoms- Access to HIV Pre-Exposure Prophylaxis (PrEP)- Access to HIV Post-Exposure Prophylaxis (PEP)- Access to sexual health information- Access to HIV and other STI testing or treatment
Substance use	Any increase or decrease in usage of substance? Possible reasons to the increase/decrease engagement in substance use? Any changes to the type of substance used during this period?	As we know Covid-19 has brought about much changes to our lives, causing us to make several adjustments to our daily routines and habits, currently is there any change in the frequency of the usage of substance in your life? How has it affected your mental health?
Relationships	Positive impact of increased time spent with partner Negative impact of increased time spent with partner (domestic abuse? Sexual violence?) Positive impact of reduced time spent with partner Negative impact of reduced time spent with partner	Due to the pandemic, several restrictions were put in place to reduce social interactions with others through reduced social gatherings outside, working from home, (for participants staying with partner): Has the increase in time spent with your partner in the same household affected your relationship in any way? Any changes to the roles you both play in the relationship? (for participants not staying with partner): Is there any changes in your relationship since both of you were unable to meet up during the circuit breaker period? How did you deal with those issues faced?
Mental health and relevant services	Exploring possibilities of mental health issues stemming from Covid-19 Exploring if Covid-19 had hindered them to seek for mental health support	This global pandemic had sent everyone into a form of social isolation, whereby we are not able to interact with others in the same way as before. How has this affected your mental well-being?- Feeling negative emotions more often?- Feelings of isolation? How has this situation created barriers for you to seek mental health support? What are the possible facilitators/plans do you think can help to address these issues?
Improvements/Suggestions		What do you think can be done to better address the issues you faced during the Covid-19 situation?- In the aspect of substance use- In the aspect of provision of sexual health services- Any miscellaneous topic
Conclusion		Once again, thank you for taking your time to join me in this session. Do you have anything else to add or share? If not, we shall end this session here. Thank you and good bye!
